# Identification, Expression, Characteristic Analysis, and Immune Function of Two *Akirin* Genes in Grass Carp (*Ctenopharyngodon idella*)

**DOI:** 10.3390/ani14162443

**Published:** 2024-08-22

**Authors:** Guokun Yang, Jianing Gu, Hao Wang, Boya Yang, Shikun Feng, Yanmin Zhang, Xindang Zhang, Xulu Chang, Jianchun Shao, Xiaolin Meng

**Affiliations:** 1 College of Fisheries, Henan Normal University, Xinxiang 453007, China; ygk5210207@126.com (G.Y.); 15101500788@163.com (J.G.); 19712550302@163.com (H.W.); YBY178628236382024@163.com (B.Y.); 2016079@htu.edu.cn (S.F.); zhang_yanmin2019@163.com (Y.Z.); zxdang1108@163.com (X.Z.); changxulu@whu.edu.cn (X.C.); 2 Engineering Technology Research Center of Henan Province for Aquatic Animal Cultivation, Henan Normal University, Xinxiang 453007, China; 3 State Key Laboratory of Mariculture Breeding, Key Laboratory of Marine Biotechnology of Fujian Province, College of Marine Sciences, Fujian Agriculture and Forestry University, Fuzhou 350002, China; shaojianchun16@mails.ucas.ac.cn

**Keywords:** grass carp, akirins, identification, expression analysis, immunologic function

## Abstract

**Simple Summary:**

Intensive aquaculture decreases immunity and increases diseases that occur in grass carp, which increases economic loss. Hence, improving grass carp immunity is an important strategy for solving the problem of intensive aquaculture. It has been reported that akirin takes part in immune responses. However, the role of akirin in grass carp is unclear. In this study, the *akirin* gene was isolated from grass carp spleen. The tissue-specific expression of *akirin* was analyzed. Akirin expression was detected by treatment with LPS, poly (I:C), and *A. hydrophila*. The immunologic function of akirin proteins was evaluated in HKLs. The findings showed that *akirin* expression was widely detected in grass carp tissues. Akirin levels were markedly induced via LPS, poly (I:C), and *A. hydrophila* stimulation in vitro and in vivo. Recombinant grass carp akirin proteins were produced by *E. coli*. The immune-related genes in HKLs were increased by treatment with recombinant akirin proteins.

**Abstract:**

Intensive aquaculture of grass carp often leads to decreased immunity and increased disease prevalence, resulting in economic losses. Improving grass carp immunity is therefore a critical strategy for addressing these challenges. Akirin reportedly participates in myogenesis, growth, and immune responses. However, its role in grass carp remains unclear. Herein, we isolated *akirins* from the spleen of grass carp and analyzed their tissue-specific expression. Akirin expression was detected following treatment with poly (I:C), LPS, and *Aeromonas hydrophila* (*A. hydrophila*). The immunological function of the akirin protein was evaluated in head kidney leukocytes (HKLs). The results revealed that the coding sequence (CDS) of *akirin1* is 570 bp, encoding 189 amino acids. There was one predicted nuclear localization signal (NLS) and two predicted α- helix domains. The CDS of *akirin2* is 558 bp, encoding 185 amino acids. There were two predicted NLSs and two predicted α-helix domains. Tissue-specific expression analysis showed that *akirins* are widely detected in grass carp tissues. *akirin1* was highly detected in the brain, kidneys, heart, spleen, and gonads, while *akirin2* was highly detected in the brain, liver, gonads, kidneys, spleen, and heart. The mRNA levels of *akirins* were promoted after treatment with poly (I:C), LPS, and *A. hydrophila*. Recombinant akirin proteins were produced in *Escherichia coli* (*E. coli*). *il-1β*, *ifnγ*, *il-6*, *tnfα*, *il-4*, *iκbα*, and *nfκb* were markedly increased in grass carp HKLs by treatment with the akirin protein. These results suggest that akirins play a role in the immunological regulation of grass carp.

## 1. Introduction

Akirin, first identified in *Drosophila*, is a conserved nuclear factor involved in organism development, myogenesis, immunity, and cellular stress responses [1,2]. Since its discovery, akirin has been identified in coelenterates, arthropods, fish, birds, and mammals [3]. In amphibians and mammals, two members of the *akirin* gene (*akirin1* and *akirin2*) are present, whereas in reptiles and birds, only *akirin2* exists [3]. It has been reported that three members of the *akirin* gene family (*akirin1*(1), *akirin2*(1), and *akirin2*(2)) are present in teleosts. Interestingly, the Salmonidae family includes eight *akirin* members [3,4]. It is indicated that the middle region of akirin proteins is less conserved, whereas the N- and C-termini are highly conserved [5]. Additionally, the conserved nuclear localization signal (NLS) (K^24^RRRC^29^) and (K^70^RRH^73^) is located at the conserved N-terminus of akirin [6,7]. Despite lacking obvious DNA-binding motifs, akirin can regulate downstream gene expression by interacting with other cofactors [8].

Akirins not only play a variety of physiological functions but are also expressed ubiquitously in the tissues of various species [7,9]. For instance, *akirin1* mRNA has been detected in the liver, brain, kidneys, lung, intestine, and testes of mice (*Mus musculus*) [10]. In aquatic animals, *akirin* genes are ubiquitously expressed in tissues [4,11,12]. However, high expression levels of *akirins* are detected in different tissues of different species. For example, *akirin* gene expression is highly detected in the hemolymph, followed by the heart, gills, and stomach of black tiger shrimp (*Penaeus monodon*) [11]. In Atlantic salmon (*Salmo salar* L.), eight *akirin* genes are highly expressed in the brain, eye, skin, kidneys, and gills [4]. Additionally, *akirin1* is highly detected in the head kidneys, liver, spleen, and muscle, and *akirin2* is highly expressed in the liver, brain, and spleen of blunt snout bream (*Megalobrama amblycephala*) [12]. Akirin expression levels also respond to pathogen stimulation, showing increased expression following challenges with *Vibrio parahaemolyticus* [11,13], *A. hydrophila* [12,14], *Edwardsiella tarda* [15], *Streptococcus iniae* [16], LPS [15,16,17], and poly I:C [15,16,17].

Although akirin, as a conserved nuclear factor, is involved in multiple physiological processes, including myogenesis [10,18], growth [3,19,20], and reproduction, immunological functions are reported in aquatic animals [11,12,14,17,21]. For example, the recombinant akirin protein increases immune-related gene expression, improving resistance to WSSV infection in red swamp crayfish (*Procambarus clarkii*) [14]. Similarly, the recombinant akirin2 protein enhances blunt snout bream resistance to *A. hydrophila* infection via the NF-κB signaling pathway [12]. Additionally, the levels of antimicrobial peptides and cytokines can be regulated in cells with an overexpression of *akirin* [11,17]. The silencing of *akirin* via RNA interference reduces *nfκb* expression in *Sogatella furcifera* [22] and increases sensitivity to WSSV and *A. hydrophila* infections in red swamp crayfish [14]. These findings highlight the crucial role of akirins in immune-related gene regulation and resistance to pathogenic infection.

Grass carp (*Ctenopharyngodon idella*) is a major freshwater species in Chinese aquaculture with high economic value. However, the expansion and development of intensive and large-scale farming has deteriorated the aquaculture environment, leading to frequent disease outbreaks in grass carp. Previous studies have reported that akirin participates in immune responses; however, its functions in grass carp are not well understood. Herein, we identified *akirin1* and *akirin2* genes from grass carp and analyzed their expression characteristics. Furthermore, we produced recombinant akirin proteins and assessed their regulatory functions on immune-related genes in vitro. To our knowledge, our study was the first to reveal the immune function of akirins in grass carp. These results can help elucidate the immune function of akirins in grass carp. These results will provide theoretical data for the application of akirins in grass carp for pathogen resistance in the future.

## 2. Materials and Methods

### 2.1. Animals

All the fish used in this study were obtained from Yanjin Fishery (Yanjin, Henan Province). The fish underwent acclimatization in indoor tanks at room temperature with recirculating water, following a cyclical light–dark photoperiod (12 h light: 12 h dark) for a minimum of two weeks. During the acclimation period, the water was half-changed every 2 days, and the water quality parameters were maintained: temperature ranged between 26 and 28 °C, pH was maintained between 7.2 and 7.5, dissolved oxygen concentration was maintained between 5.5 and 6.2 mg/L, and total ammonia nitrogen level <0.02 mg/L. This study was conducted in strict compliance with the guidelines stipulated in the Animal Ethics Procedures and Guidelines of the People’s Republic of China. Approval for all animal protocols was obtained from the Animal Administration and Ethics Committee of Academic Committee of Henan Normal University (HNSD-2024-08-27).

### 2.2. Isolation of Grass Carp Akirin and Tissue Expression Characteristic Analysis

Reverse transcriptase-PCR (RT-PCR) was employed for the cloning of the *akirin* coding sequences (CDS). The predicted sequences of grass carp *akirin1* (GEUQ01031657) and *akirin2* (GEUQ01062159) were obtained from the transcriptome shotgun assembly database at the National Center for Biotechnology Information through a sequence alignment with zebrafish (*Danio rerio*) *akirin1* (NM 001113800.2) and *akirin2* (BC097074.1) sequences. Total RNA was extracted from the spleen of grass carp, and the first-strand cDNA was synthesized by reverse transcription kit (Takara, Dalian, China). Specific primers designed based on the akirin sequences were synthesized for the purpose of gene cloning. The PCR cycling conditions were as follows: 94 °C for 3 min; followed by 35 cycles of 94 °C for 30 s, 56 °C for 30 s, 72 °C for 1 min; 72 °C, 5 min; and 12 °C for infinity. The SignalP 5.0 server (http://www.cbs.dtu.dk/services/SignalP/ (accessed on 10 May 2023)) was utilized to analyze the signal peptide of akirins. The spatial structure of grass carp akirins was examined using the Swiss model (https://swissmodel.expasy.org/ (accessed on 12 May 2023)). The PSORT (https://www.genscript.com/psort.html (accessed on 15 May 2023)) was used to investigate the nuclear localization signal (NLS) motif of akirins, and the sequence alignment of akirins was evaluated using ClustalW2 (http://www.ebi.ac.uk/Tools/msa/clustalw2/ (accessed on 15 May 2023)). A phylogenetic tree of akirins was constructed with MEGAX using the neighbor-joining method (bootstrap phylogeny test, 2000 replicates).

For the tissue-specific expression analysis, grass carp (300–350 g) were used to evaluate *akirin* expression levels in different tissues. The fish were anesthetized with MS222 and subsequently decapitated to obtain the tissues. Total RNA was isolated from the collected tissues, including telencephalon, mesencephalon, cerebellum, hypothalamus, pituitary, head kidney, kidney, heart, liver, spleen, foregut, midgut, hindgut, fat, muscle, gonad, and gill. The quantification of akirin mRNA levels was conducted using real-time PCR.

### 2.3. CIK Cells Cultured and Effect of Poly (I:C) and LPS on Akirin Levels

The grass carp kidney (CIK) cell line, preserved in our laboratory, was cultured in M199 medium (Gibco, CA, USA) with 100 IU/mL of penicillin, 100 mg/mL of streptomycin, and 10% fetal bovine serum (FBS). The cells were maintained at 28 °C in a 5% CO_2_ environment. CIK cells were digested by 0.25% trypsin (Thermo Fisher, Wilmington, DE, USA) and cultured in 24-well plates. Following a 24-h incubation period, the culture medium was replaced, and the cells were treated with different concentrations of LPS (Sigma, St. Louis, MO, USA) at 5, 10, and 20 μg/mL or poly (I:C) (Sigma, St. Louis, MO, USA) at 1, 5, and 25 μg/mL. RNAiso Plus was used to lyse CIK cells post-treatment with LPS or poly (I:C) for 6 and 12 h. Subsequently, cell samples were collected for RNA extraction, and *akirin* levels were quantified using real-time PCR.

### 2.4. Isolation of Primary HKLs and Treatment with LPS and Poly (I:C)

HKLs were isolated using discontinuous density gradient centrifugation, as described previously [23]. Briefly, grass carp were sterilized with 75% ethanol and then euthanized. The head kidney was collected, washed, and pressed in RPMI-1640 medium. Subsequently, the cell suspension was filtered through a 200 μm nylon mesh and centrifuged on Histopaque^®^-1083 (Sigma-Aldrich, St. Louis, MO, USA, density 1.083 kg/L) at 4 °C, 500 g for 25 min. The isolated leukocytes were then washed twice with PBS, resuspended in RPMI-1640 medium supplemented with 10% FBS, and seeded at a density of 2 × 10^6^ cells per well in 24-well plates. The cells were then incubated at 28 °C under 5% CO2 and saturated humidity. Following overnight culture, the medium was replaced with fresh RPMI-1640, and the leukocytes were exposed to 25 μg/mL ploy (I:C) or 20 μg/mL LPS for 3, 6, 12, 24, and 48 h. At the conclusion of the experiment, the cells were lysed using RNAiso Plus for RNA extraction, and the expression of *akirins* was assessed through real-time PCR.

### 2.5. Stimulation with Poly (I:C) and Challenge with A. hydrophila

*A. hydrophila* challenge experiment was performed as described in previous study [24]. The preserved strain of *A. hydrophila* from our laboratory was utilized. The bacteria were cultivated in LB medium until reaching an OD600 of 0.6. Subsequently, the bacteria solution underwent centrifugation, was washed twice with PBS, and resuspended with 0.65% saline at a concentration of 1.4 × 10^6^ CFU/mL.

Following a two-week acclimatization period, grass carp in good health were allocated into three distinct groups: control, *A. hydrophila,* and poly (I:C) groups. The fish received injections of 0.65% saline (control group), *A. hydrophila* (8.6 × 10^5^ CFU/fish), or poly (I:C) (5 μg/g body weight). Subsequently, at 3, 6, 12, 24, and 48 h post-injection, the fish were anesthetized (MS222, 100 mg/L) and decapitated. Tissue samples from the liver, spleen, head kidney, and kidney were collected for RNA extraction. The expression of *akirins* was assessed using real-time PCR.

### 2.6. Production of Recombinant Akirin1 and Akirin2 Protein in Escherichia coli

Recombinant protein production was performed using a previously described method [25]. Recombinant expression plasmids (pET21a-akirin1 and pET21a-akirin2) were constructed using specific primers (Table 1). The *E. coli* BL21 (DE3) strain (Solarbio, Beijing, China) was used to produce the recombinant protein. A positive clone was cultured in LB-ampicillin (100 μg/mL) medium at 37 °C with agitation at 180 rpm until the OD600 reached 0.5–0.7. Induction of recombinant protein production was achieved using Isopropyl-β-D-thiogalactoside (1 mM). Following a 4 h induction period, the recombinant bacteria were subjected to centrifugation, ultrasonication, and subsequent centrifugation. The supernatants containing recombinant akirin1 and akirin2 proteins were purified using a Ni-NTA resin column (GE, ConnecticutUSA), dialyzed in PBS buffer, and concentrated through ultrafiltration. To eliminate endotoxin contamination, a ToxinEraser Endotoxin Removal Kit (GenSript, Piscataway, NJ, USA) was employed. The quantification of the purified proteins was performed using the BCA assay (Solarbio, Beijing, China).

### 2.7. Effect of Akirin1 and Akirin2 on the Expression Levels of Immune-Related Genes in HKLs

HKLs were isolated as described in Section 2.4 and seeded at a density of 2 × 10^6^ cells per well in 24-well plates. The cells were then incubated at 28 °C under 5% CO_2_ and saturated humidity. Following overnight culture, the medium was replaced with fresh RPMI-1640. Leukocytes were treated with purified akirin1 or akirin2 protein at concentrations of 0, 10, 100, and 1000 ng/mL for 12 h. RNAiso Plus was used to lyse the leukocytes for RNA extraction, and the expression of immune-related genes was assessed using real-time PCR.

### 2.8. Procedures for RNA Extraction, cDNA Synthesis, and Real-Time PCR

RNAiso Plus was utilized for the extraction of total RNA. The concentration of total RNA was determined using a UV spectrophotometer (Nanodrop 2000, Thermo, Wilmington, DE, USA). Subsequently, 1 μg of total RNA underwent digestion with gDNA Eraser at 42 °C for 2 min to remove genomic DNA. The first-strand cDNA was then synthesized using PrimeScript RT reagent kit (PrimeScript RT reagent kit with gDNA Eraser, Takara). The resulting first-strand cDNA served as the template for real-time PCR, with the primer sequences detailed in Table 1. Real-time PCR was performed using SYBR green qPCR mix (Bimake, Shanghai, China) on the LightCycler 480 II Sequence Detection System (Roche, Rotkreuz, Switzerland) following the manufacturer’s protocol. The real-time PCR reaction was carried out in a total volume of 10 μL under the following conditions: 95 °C for 5 min; followed by 40 cycles of 95 °C for 15 s, 56 °C for 15 s, and 72 °C for 30 s. Melting curve analysis of the amplification products was performed to assess the specificity of the PCR products at the conclusion of each PCR run, with the melting curves of the target genes exhibiting single peaks. 18S rRNA and β-actin were used as the internal reference genes, demonstrating stability across various experimental conditions. The gene expression levels were normalized to 18S rRNA or β-actin, and the results were analyzed using the comparative Ct method [26].

### 2.9. Statistical Analysis

The data were represented as mean ± S.E.M. Normality and homogeneity of variance were checked using the Shapiro–Wilk and Levene’s tests. Statistical analysis was performed on SPSS version 18.0 (SPSS Inc., Chicago, IL, USA). The data were subjected to one-way ANOVA, followed by Duncan’s multiple comparison tests as a post hoc analysis to evaluate differences among the groups. Statistical significance was considered at *p* < 0.05, *p* < 0.01 and *p* < 0.001.

## 3. Results

### 3.1. Identification and Sequence Analysis of Grass Carp Akirin

**Figure 1:**
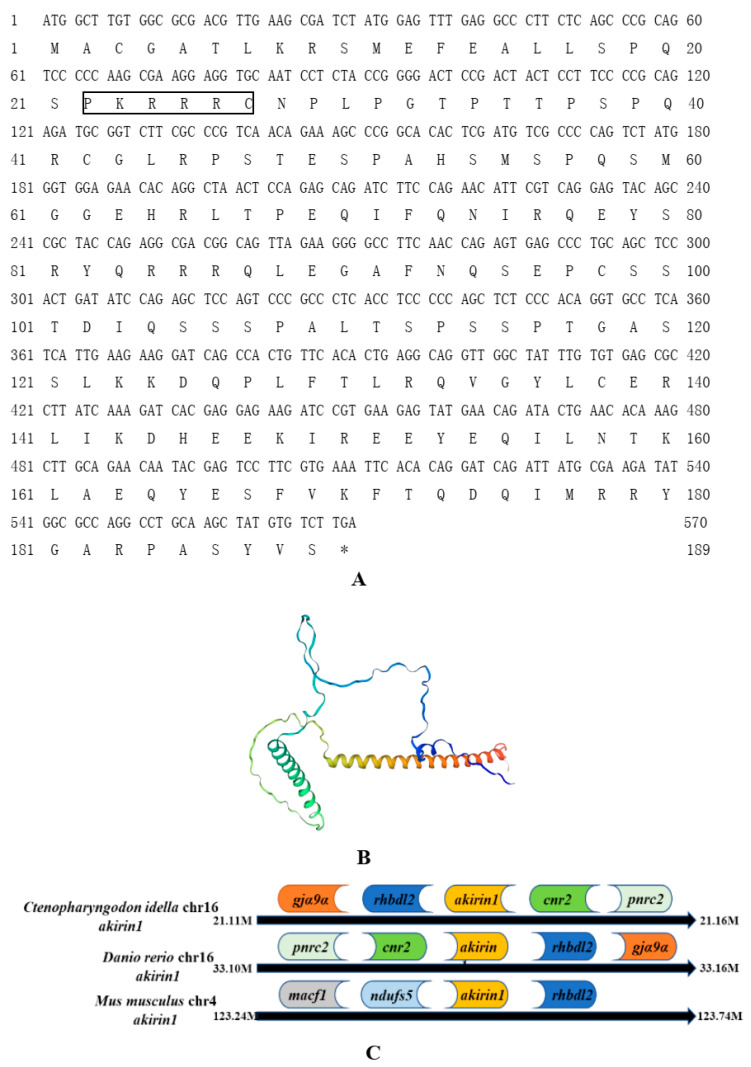
(**A**) The cDNA and deduced amino acids of grass carp akirin1. Box represents NLS motif; the * represents the stop codon. (**B**) The predicted three-dimensional structure of grass carp akirin1. The model W5KQL2.1.A (*Astyanax mexicanus* akirin1) was used as the reference model for the predicted spatial structure. (**C**) Gene synteny and chromosomal location analysis of *akirin1*. The genes adjacent to *akirin1* loci are shown on grass carp and zebrafish chromosome 16 and mouse chromosome 4.

**Figure 2:**
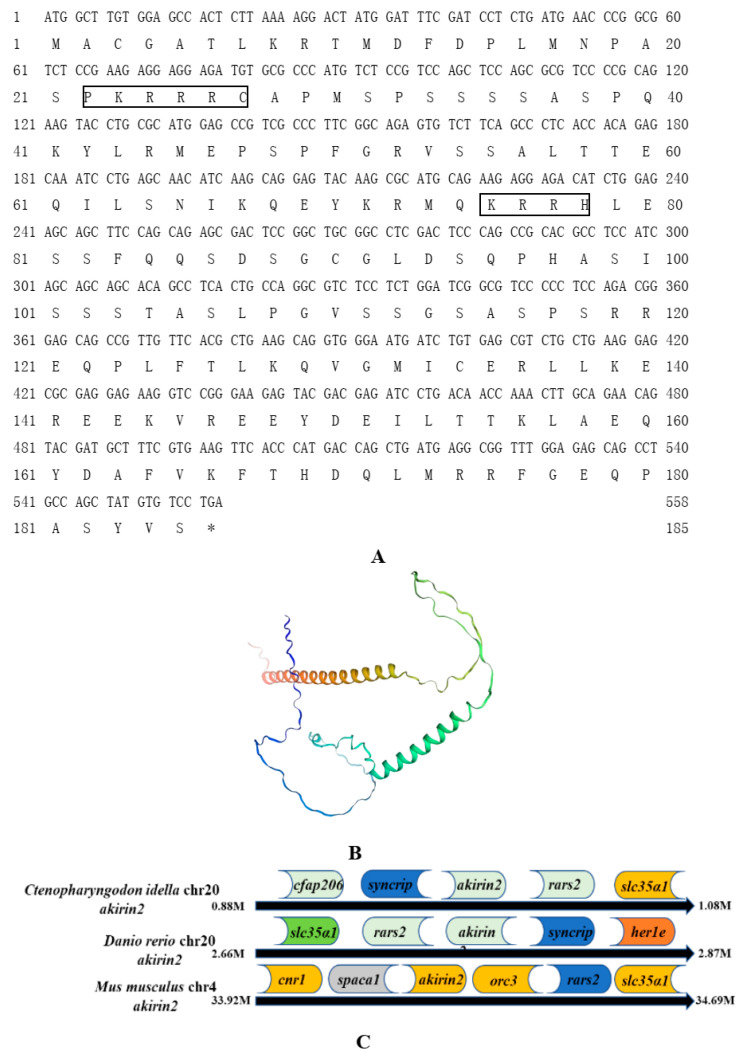
(**A**) The cDNA and deduced amino acids of grass carp akirin2. Box represents NLS motif; the * represents the stop codon. (**B**) The predicted three-dimensional structure of grass carp akirin2. The model A8YXY8.1.A (*Bos taurus* akirin2) was used as the reference model for the predicted spatial structure. (**C**) Gene synteny and chromosomal location analysis of *akirin2*. The genes adjacent to *akirin2* loci are shown on grass carp and zebrafish chromosome 20 and mouse chromosome 4.

**Figure 3:**
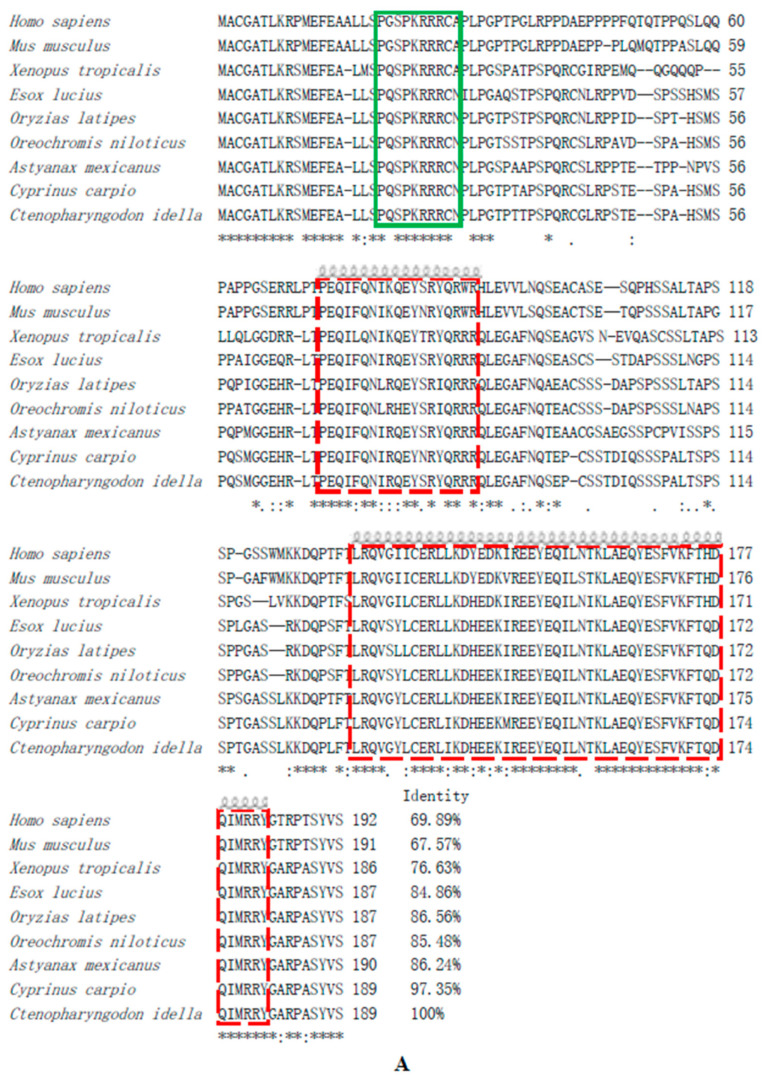

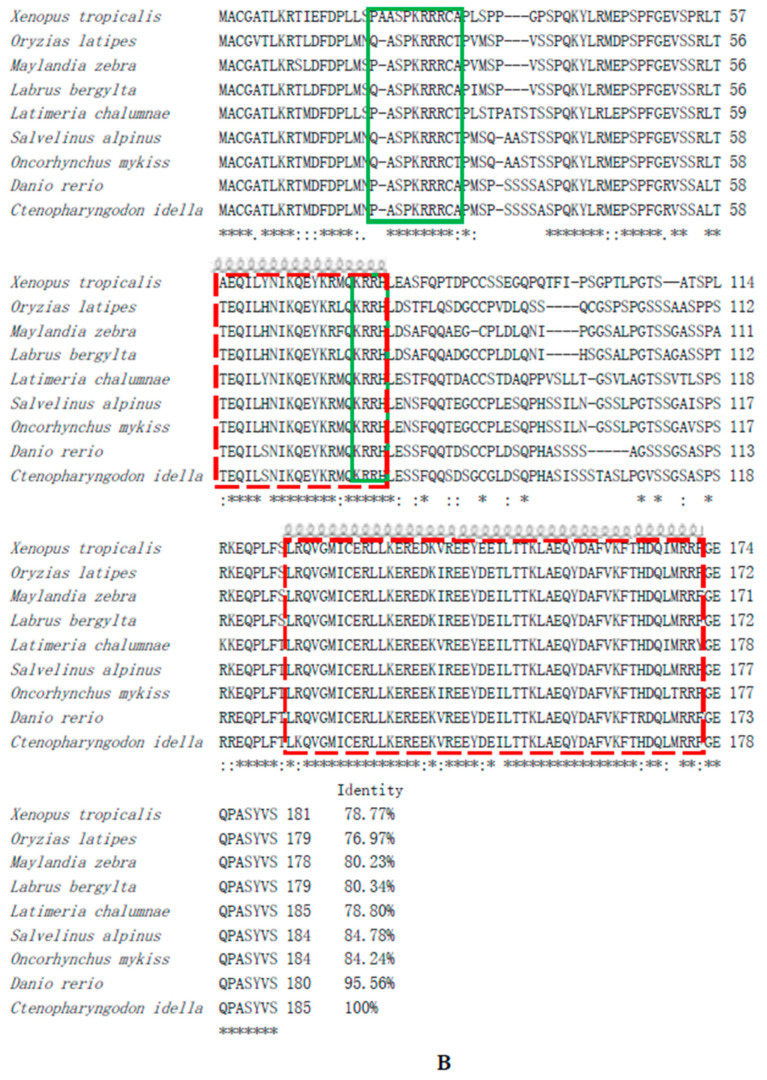
Sequence alignment of grass carp akirin1 (**A**) and akirin2 (**B**) with other species. The identical amino acids are noted by (*), and the highly and less conserved amino acids are noted by (:) and (.), respectively. The green box represents NLS motif. The red box represents α-helix motif. The identity of each sequence compared is indicated at the end of the sequences.

**Figure 4:**
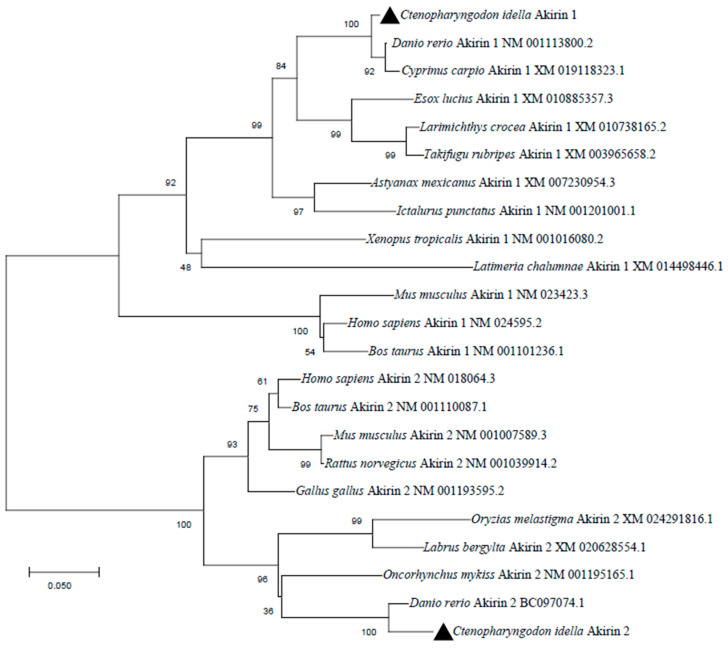
Phylogenetic tree based on amino acid alignment for akirin1 and akirin2 in different species.

According to sequence analysis, the results showed that genes neighboring the loci of grass carp akirin1 and akirin2 exhibited a conserved chromosomal synteny with those of zebrafish and mice. Additionally, the positioning of the neighboring genes to grass carp *akirin1* and akirin2 corresponded with that of zebrafish *akirin1* and *akirin2* (Figure 1C and Figure 2C). The grass carp *akirin1* and akirin2 were determined to be 570 bp and 558 bp, encoding 189 and 185 amino acids, respectively (Figure 1A and Figure 2A). The predicted molecular weights of akirin1 and akirin2 were calculated to be 21.46 kDa and 20.80 kDa, with theoretical isoelectric points of 9.18 and 9.25, respectively. One predicted NLS sequence (P^18^QSPKRRRCN^28^) and two predicted α- helix domains were identified in the akirin1 protein (Figure 1A). In addition, two predicted NLS sequences (P^18^ASPKRRRCA^28^, K^75^RRH^78^) and two predicted α- helix domains were found in the cloned akirin2 (Figure 2A). The results of the sequence alignment indicated a high sequence identity among different akirin1 proteins (greater than 84.86%) and akirin2 proteins (greater than 76.79%) (Figure 3). The phylogenetic analysis revealed that the cloned akirin1 and akirin2 clustered within the akirin1 and akirin2 subgroup along with other fish species (Figure 4). Furthermore, the structural comparison showed that the spatial structure of grass carp akirin1 shared 84.80% identity with the model W5KQL2.1.A (*Astyanax mexicanus* akirin1) (Figure 1B), while the spatial structure of grass carp akirin2 exhibited 81.52% identity with the model A8YXY8.1.A (*Bos taurus* akirin2) (Figure 2B).

### 3.2. Analysis of the Tissue Expression Characteristics of Akirins in Grass Carp

**Figure 5:**
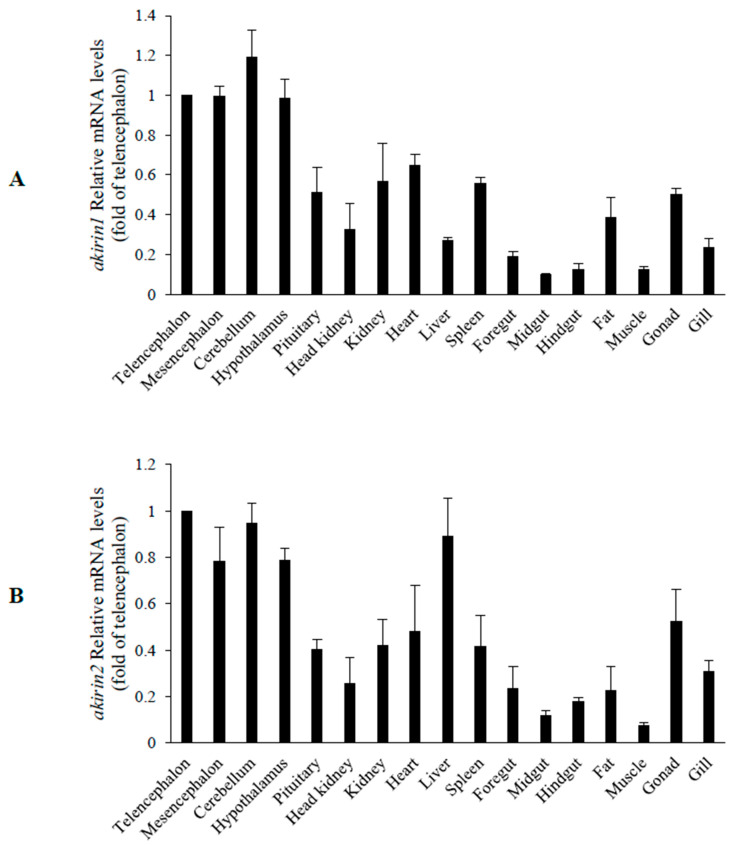
Tissue expression analysis of *akirin1* (**A**) and *akirin2* (**B**) in grass carp. The expression levels were evaluated by real-time PCR. All data are represented as the mean ± S.E.M. (n = 3).

The real-time PCR results indicated the widespread presence of *akirin1* mRNA in different tissues of grass carp. Elevated levels of *akirin1* mRNA were observed in the brain, kidney, heart, spleen, and gonad (Figure 5A). Likewise, *akirin2* mRNA was broadly detected in different tissues of grass carp, exhibiting high expression levels in the brain, liver, gonad, kidney, spleen, and heart (Figure 5B).

### 3.3. Effect of Poly (I:C) and LPS on Akirin mRNA Expression in CIK Cells and HKLs

**Figure 6:**
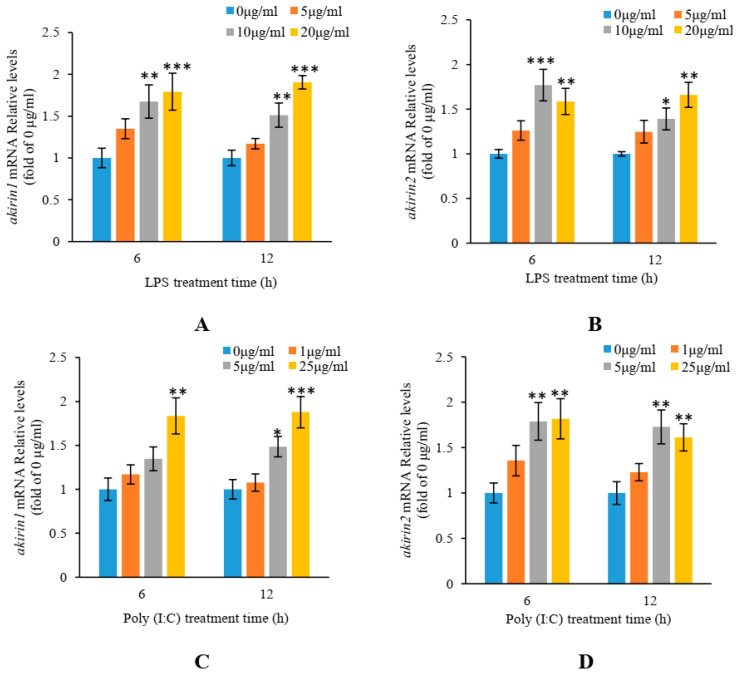
Effect of LPS and poly (I:C) on *akirin1* (**A**,**C**) and *akirin2* (**B**,**D**) expression in CIK cells. The CIK cells were digested by 0.25% trypsin, and seeded into 24-well culture plates. The medium was changed and treated with 5, 10 and 20 μg/mL LPS or 1, 5 and 25 μg/mL ploy (I:C) after CIK cells culture for 24 h. The CIK cells were lysed by RNAiso Plus after treatment with LPS or ploy (I:C) for 6 and 12 h. All data are shown as mean ± S.E.M. (n = 5–6). The data were analyzed using one-way ANOVA. *, *p* < 0.05; **, *p* < 0.01; ***, *p* < 0.001.

**Figure 7:**
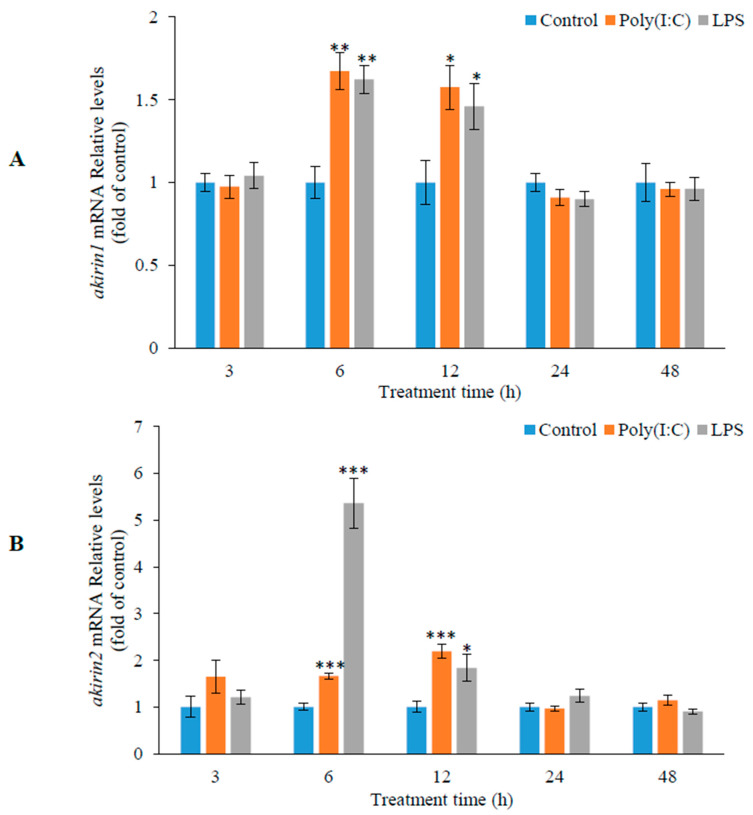
Effect of LPS and poly (I:C) on *akirin1* (**A**) and *akirin2* (**B**) expression in HKLs. The HKLs were isolated by discontinuous density gradient centrifugation. The cells were seeded in 24 well cell cultured plates with density of 2 × 10^6^ cells/well. After overnight culture, the cells were treated with 20 μg/mL LPS or 25 μg/mL ploy (I:C) for 3, 6, 12, 24 and 48 h. At end of the experiment, the cells were lysed by RNAiso Plus for RNA extraction and detecting the *akirin* expression. All data are shown as mean ± S.E.M. (n = 5–6). The data were analyzed using one-way ANOVA. *, *p* < 0.05; **, *p* < 0.01; ***, *p* < 0.001.

In Figure 6, the mRNA levels of *akirin1* and *akirin2* in CIK cells were assessed using real-time PCR. The results revealed that the expression of *akirin1* and *akirin2* was upregulated following treatment with 10 and 20 μg/mL LPS. Additionally, the expression of *akirin1* and *akirin2* was significantly increased after exposure to poly (I:C) for 6 and 12 h. Moreover, the mRNA levels of *akirin1* and *akirin2* in HKLs were notably elevated following treatment with 20 μg/mL LPS and 25 μg/mL poly (I:C) for 6 and 12 h (Figure 7).

### 3.4. Effect of Poly (I:C) Stimulation and A. hydrophila Challenge on the Expression of Akirin

**Figure 8:**
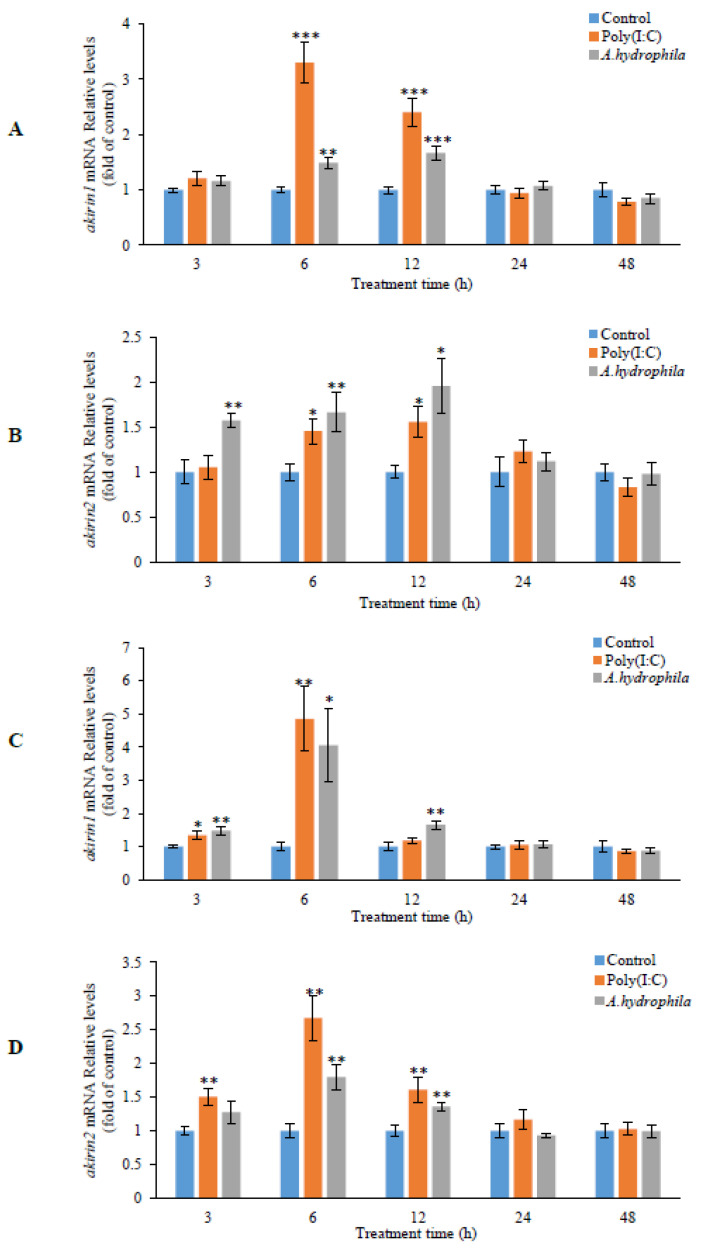

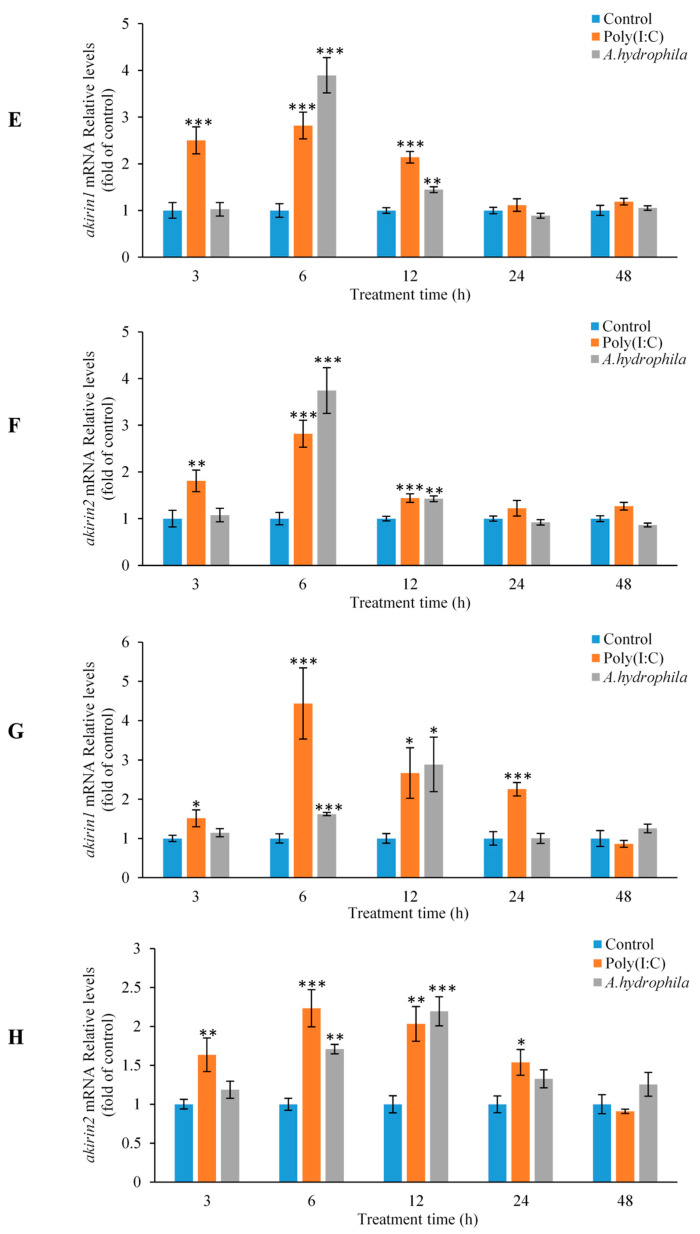
Effect of *A. hydrophila* challenge and poly (I:C) stimulation on *akirin1* and *akirin2* expression by intraperitoneal injection. The fish were intraperitoneally injected with 0.65% saline, *A. hydrophila* or poly (I:C), respectively. At 3, 6, 12, 24 and 48 h post injection, the fish were anesthetized and decapitated. The *akirin* expression was analyzed in liver (**A**,**B**), spleen (**C**,**D**), kidney (**E**,**F**), and head kidney (**G**,**H**) by real-time PCR. All data are shown as mean ± S.E.M. (n = 7–8). The data were analyzed using one-way ANOVA. *, *p* < 0.05; **, *p* < 0.01; ***, *p* < 0.001.

Upon challenge with *A. hydrophila*, the real-time PCR results indicated a significant increase in the expression of *akirin1* and *akirin2* in the head kidney, kidney, spleen, and liver at 3, 6, and 12 h (Figure 8). Additionally, the expression of *akirin1* and *akirin2* was notably induced by poly (I:C) stimulation in the head kidney, liver, spleen, and kidney from 3 to 24 h (Figure 8).

### 3.5. Recombinant Akirin Proteins Were Produced by E. coli

**Figure 9:**
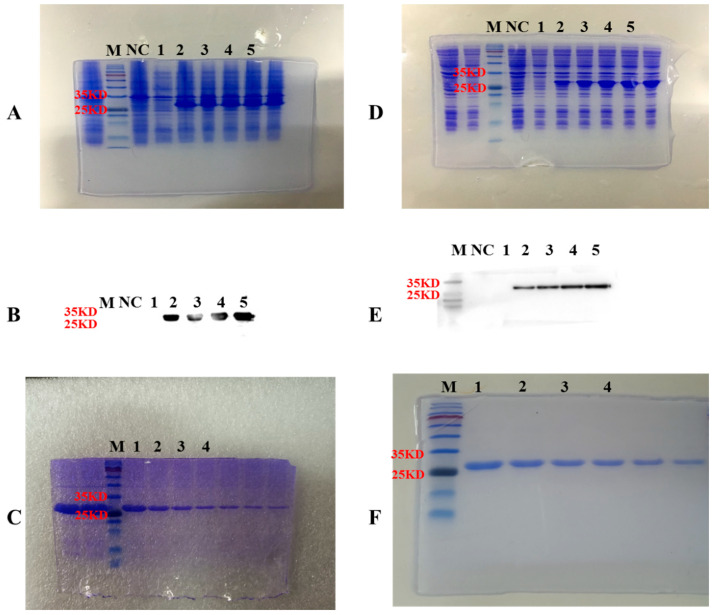
Recombinant grass carp akirin1 and akirin2 protein production. (**A**,**D**). Recombinant grass carp akirin1 (**A**) and akirin2 (**D**) expression strain construction and screening. M, protein marker; NC, mock vector; 1, induced expression for 0 h; 2–5, induced expression for 1, 2, 3, 4 h. (**B**,**E**). Identification of recombinant grass carp akirin1 (**B**) and akirin2 (**E**) protein by Western blotting. The target protein was identified by Western blot with His-tagged monoclonal antibody. M, protein marker; NC, mock vector; 1, induced expression for 0 h; 2–5, induced expression for 1, 2, 3, 4 h. (**C**,**F**). Recombinant grass carp Akirin1 (**C**) and Akirin2 (**F**) affinity purification. M, protein marker; 1–4, different fraction eluted purified protein.

The recombinant grass carp akirin1 and akirin2 were expressed using *E. coli*. As shown in Figure 9, the SDS-PAGE and Western blot results demonstrated that the molecular weights of recombinant grass carp akirin1 and akirin2 were as expected. Following purification, the proteins exhibited a single band, indicating their suitability for further experimental procedures (Figure 9).

### 3.6. Effect of Akirin1 and Akirin2 on Gene Expression in HKLs

**Figure 10:**
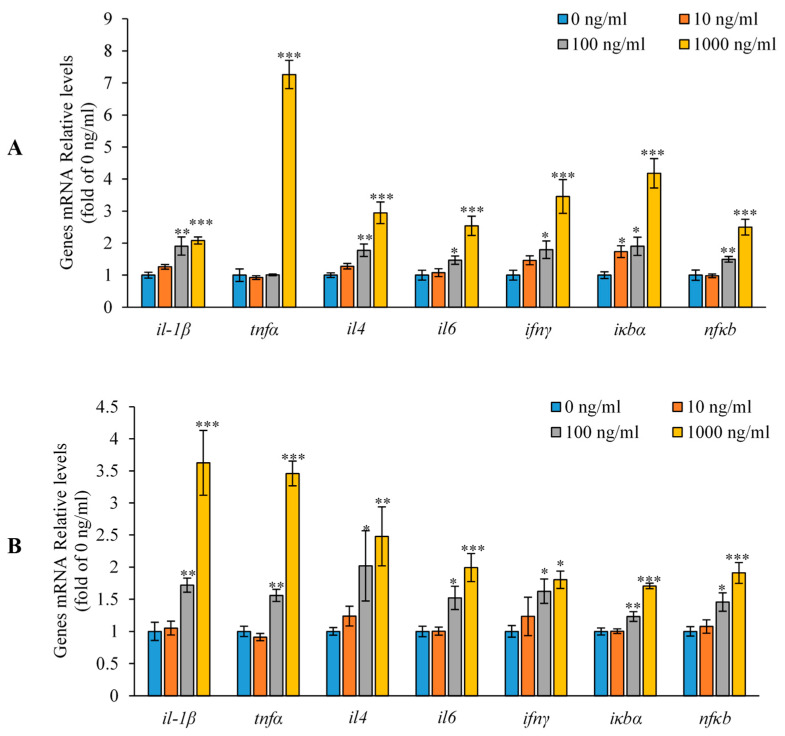
Effect of akirin1 and akirin2 protein on immune related gene expression of HKLs. (**A**), akirin1 protein treatment; (**B**), akirin2 protein treatment. The HKLs were isolated by discontinuous density gradient centrifugation. The cells were seeded in 24 well cell cultured plates with density of 2 × 10^6^ cells/well. After overnight culture, the cells were treated with akirin1 and akirin2 protein (0, 10, 100, 1000 ng/mL) for 12 h. At end of the experiment, the cells were lysed by RNAiso Plus for RNA extraction and detecting immune related genes expression. All data are shown as mean ± S.E.M. (n = 5–6). The data were analyzed using one-way ANOVA. *, *p* < 0.05; **, *p* < 0.01; ***, *p* < 0.001.

As shown in Figure 10, the real-time PCR results revealed that the mRNA levels of *il-1β*, *il-4*, *il-6*, *ifnγ,* and *nfκb* were significantly upregulated following treatment with 100 and 1000 ng/mL of akirin1. Furthermore, the expression of *iκbα* mRNA was induced by 10, 100, and 1000 ng/mL of akirin1. However, *tnfα* mRNA levels were notably increased only with 1000 ng/mL of akirin1. Similarly, the mRNA levels of *il-1β*, *ifnγ*, *il-4*, *tnfα*, *il-6*, *iκbα,* and *nfκb* mRNA were markedly elevated with 100 and 1000 ng/mL of akirin2 treatment (Figure 10).

## 4. Discussion

Since the first report about *akirins* in 2008, they have been associated with a variety of physiological functions [27], encompassing carcinogenesis [8], myogenesis [10,18,28], reproduction [29], growth [3,19,20], and innate immune responses [1,21]. To explore the roles of akirin in grass carp, *akirin1* and *akirin2* genes were isolated from the grass carp spleen. The conserved N-terminal NLS is a known functional motif in akirins [1,5], and similarly, the conserved N-terminal predicted NLS was identified in the isolated grass carp akirins. Additionally, another NLS was observed in grass carp akirin2. Previous studies have reported the detection of two NLS motifs in akirin2 isolated from different species, such as black tiger shrimp [11], Chinese mitten crab [30], amphioxus [31], and blunt-snout bream [12]. The NLS motif facilitates the translocation of akirins into the cell nucleus [6]. For instance, the akirin1 protein is present in both the cytoplasm and nucleus of C2C12 myoblasts [32]. However, amphioxus akirin2, with two NLS motifs, was exclusively localized in the nucleus of HEK293T cells [31]. Therefore, the potential biological function of the NLS motif in grass carp warrants further exploration. The cloned akirin proteins herein contain two α-helix motifs. It has been reported that α-helix structures play a role in maintaining the structure and function of akirins [27]. Despite the structural similarities between grass carp akirins and identified akirins, the specific biological functions of NLS and α-helix motifs in grass carp akirins remain ambiguous. Further investigation is required to determine whether the functions of these motifs in grass carp are consistent with previous reports or if they exhibit distinct activities.

Previous research has indicated the presence of *akirin* mRNA in various tissues [7,9]. The widespread distribution of *akirins* across different tissues is associated with their diverse physiological functions. High levels of akirins have been observed in the brain, kidney, liver, gonad, spleen, and heart of grass carp. Similarly, either *akirin1* or *akirin2* has been identified in the brain, kidney, liver, and testes of mice [10] and Chinese loach [17]. Furthermore, in blunt-snout bream [12] and croaker [3], *akirins* were found to be significantly expressed in the liver, spleen, brain, head kidney, and kidney. These findings suggest that the expression of *akirin* in nonimmune-related tissues is a common occurrence. The notable presence of *akirins* in nonimmune-related tissues may be linked to the functions such as reproduction, growth, myogenesis, and other non-immunological processes.

The expression levels of *akirin* are influenced by immune stimulation [6,11]. In this study, *akirin* expression was induced by poly (I:C), LPS, and *A. hydrophila* stimulation both in vitro and in vivo. Similarly, in Hong Kong oysters [33], silkworms [34], and black tiger shrimp [11], *akirin* expression was upregulated following poly (I:C), LPS, and pathogenic stimulation. Moreover, in Chinese mitten crab [28], red swamp crayfish [14], blunt-snout bream [12], Chinese loach [17], rock bream [15], and yellowtail clownfish (*Amphiprion clarkii*) [35], *akirin* mRNA levels were enhanced by LPS, *A. hydrophila,* and poly (I:C) stimulation. Poly (I:C), LPS, and *A. hydrophila* are commonly used as viral and bacterial mimics in the immunological research of aquatic animals. These results indicate that akirins play a role in immune regulation in response to viral and bacterial infections.

Akirins have been reported to play a significant role in regulating the expression of immune factors [12,14,17,30]. Treatment with recombinant akirin1 and akirin2 resulted in a notable increase in levels of *tnfα*, *il-1β*, *il-4*, *il-6*, *ifnγ,* and *iκbα*. The overexpression of the Chinese loach *akirin2* gene led to the upregulation of *tnfα*, *il-1βil-4* mRNA levels in ZF4 cells [17]. Moreover, the overexpression of *akirin2* was found to elevate *il-6* levels in mice [1] and pigs (*Sus scrofa*) [36]. The recombinant akirin protein was also shown to enhance mRNA levels of immune-related genes, thereby enhancing the resistance to WSSV infection in red swamp crayfish [14]. Furthermore, treatment with recombinant akirin2 protein induced the expression of the antimicrobial peptide, boosting immunity to *A. hydrophila* infection in blunt-snout bream [12]. While there are no reports on akirin1 regulating tnfα expression, the present study demonstrated a significant increase in *tnfα* expression levels with 1000 ng/mL of recombinant akirin1 protein. However, the *tnfα* expression level was induced by 100 and 1000 ng/mL of recombinant akirin2 protein. It is speculated that the *tnfα* expression may be more responsive to akirin2 than akirin1 in grass carp. In conclusion, akirin levels are upregulated in response to pathogen infection in aquatic animals, and the induced akirins enhance resistance to pathogen infection by upregulating immune-related gene levels.

It has been reported that akirins exert their physiological function by mediating the NF-κB signaling pathway [2,12,30,37]. In this study, *nfκb* expression increased following akirin treatment. Previous studies have demonstrated that the overexpression of *akirin2* can elevate *nfκb* expression in ZF4 and human cells [17,38]. Moreover, akirin2 increased antimicrobial peptide levels through the NF-κB signaling pathway to combat *A. hydrophila* infection in blunt-snout bream [12]. Additionally, luciferase assays revealed that the NF-kB luciferase reporter was activated in cells overexpressing *akirins* [16,30,33]. These results indicate that the NF-κB signaling pathway plays a role in immunological regulation by akirins. Although *nfκb* expression levels were induced by akirins, further research is needed to explore the regulatory mechanism of akirins in increasing immune-related gene levels and enhancing resistance to pathogen infection through mediating the NF-κB signaling pathway.

## 5. Conclusions

Two *akirin* genes were identified from grass carp. Akirin expression was widely detected in grass carp tissues, with high expression levels in the brain, liver, kidney, heart, spleen, and gonad. Additionally, the *akirin* expression levels were markedly induced via LPS, poly (I:C), and *A. hydrophila* stimulation in vitro and in vivo. Recombinant grass carp akirin proteins were produced by *E. coli*. The immune-related genes in HKLs were increased by treatment with recombinant akirin proteins. In conclusion, akirins take part in the regulation of immune-related gene expression and promote resistance to pathogen infection of grass carp. These results will provide theoretical data for the application of akirins in aquaculture for pathogen resistance in the future.

## Figures and Tables

**Table 1:** Primers used in this study.

Genes	Sequence (5′→3′)	Accession No.	PCR Efficiency
akirin1-ORF-F	ATGGCTTGTGGCGCGACGT		
akirin1-ORF-R	TCAAGACACATAGCTTGCA		
akirin2-ORF-F	ATGGCTTGTGGAGCCACT		
akirin2-ORF-R	TCAGGACACATAGCTGGCA		
rAkirin1-F	CGGAATTCCATCATCATCATCATCATATGGCTTGTGGCGCGACG		
rAkirin1-R	ATAGTTTAGCGGCCGCTCAAGACACATAGCTTGC		
rAkirin2-F	CGGAATTCCATCATCATCATCATCATATGGCTTGTGGAGCCACT		
rAkirin2-R	ATAGTTTAGCGGCCGCTCAGGACACATAGCTGGC		
*akirin1*-qRT-F	GTCTTCGCCCGTCAACA		1.912
*akirin1*-qRT-R	TAACTGCCGTCGCCTCT		
*akirin2*-qRT-F	CGACTCCCAGCCGCACGCCT		1.934
*akirin2*-qRT-R	CCTTCAGCAGACGCTCACAG		
*ifnγ*-qRT-F	TGTTTGATGACTTTGGGATG	JX657682	1.918
*ifnγ*-qRT-R	TCAGGACCCGCAGGAAGAC		
*tnfα*-qRT-F	CGCTGCTGTCTGCTTCAC	HQ696609	1.951
*tnfα*-qRT-R	CCTGGTCCTGGTTCACTC		
*il-1β*-qRT-F	AGAGTTTGGTGAAGAAGAGG	JQ692172	1.944
*il-1β*-qRT-R	TTATTGTGGTTACGCTGGA		
*il-6*-qRT-F	CAGCAGAATGGGGGAGTTATC	KC535507.1	2.036
*il-6*-qRT-R	CTCGCAGAGTCTTGACATCCTT		
*il-4*-qRT-F	CTACTGCTCGCTTTCGCTGT	KT445871	2.015
*il-4*-qRT-R	CCCAGTTTTCAGTTCTCTCAGG		
*nfκb*-qRT-F	GAAGAAGGATGTGGGAGATG	KJ526214	1.964
*nfκb*-qRT-R	TGTTGTCGTAGATGGGCTGAG		
*iκbα*-qRT-F	TCTTGCCATTATTCACGAGG	KJ125069	1.998
*iκbα*-qRT-R	TGTTACCACAGTCATCCACCA		
*18s*-qRT-F	ATTTCCGACACGGAGAGG	EU047719	1.941
*18s*-qRT-R	CATGGGTTTAGGATACGCTC		
*β-actin*-qRT-F	GGCTGTGCTGTCCCTGTA	M25013	2.003
*β-actin*-qRT-R	GGGCATAACCCTCGTAGAT		

## Data Availability

The data that support the findings of this study are available from the corresponding author upon reasonable request.
